# Analytical Performance of the RIDASCREEN^®^ Hantavirus Puumala IgG/IgM ELISA Assay

**DOI:** 10.3390/v12020226

**Published:** 2020-02-18

**Authors:** Melissa Depypere, Katrien Lagrou, Marjan Van Esbroeck, Els Houben, Marc Van Ranst

**Affiliations:** 1 National Reference Center for Hantavirus, Department of Laboratory Medicine, University Hospitals Leuven, Herestraat 49, 3000 Leuven, Belgium; Katrien.lagrou@uzleuven.be (K.L.); els.houben@uzleuven.be (E.H.); marc.vanranst@uzleuven.be (M.V.R.); 2 Institute of Tropical Medicine Antwerp, Kronenburgstraat 43/3, 2000 Antwerp, Belgium; mvesbroeck@itg.be

**Keywords:** Hantavirus, Orthohantavirus, Puumalavirus, Ridascreen

## Abstract

The National Reference Center for Hantavirus in Belgium is currently using the Hantavirus IgM/IgG ELISA Progen kit (Heidelberg, Germany) for the detection of the most prevalent Hantavirus in Western Europe, Puumala virus (PUUV). Two commercially available PUUV kits were compared: Progen and RIDASCREEN^®^ Hantavirus Puumala IgM/IgG ELISA assay (Darmstadt, Germany). Methods: The sensitivity was evaluated with a panel of 68 samples from patients with an acute infection (*n* = 44) or a past infection (*n* = 24). Specificity was evaluated with a panel of 62 samples from patients with potentially false borderline results (*n* = 7) (no seroconversion), seronegative samples (*n* = 25) and potentially cross reacting samples (*n* = 30). Discordances were resolved by immunoblot. Substantial agreement was calculated using Cohen kappa coefficient. Results: The RIDASCREEN^®^ kit showed a higher specificity (IgM: 94.3%; IgG: 94.4%) than the Progen kit (IgM: 77.0% IgG: 93.0%). The sensitivity for IgM ELISA was 100% for both assays. IgG sensitivity was, respectively, 98.3% and 100% for Progen and RIDASCREEN^®^. A Cohen kappa coefficient of 0.76 and 0.90 was found between Puumala IgM and IgG, respectively. Conclusions: This study showed a higher specificity for the RIDASCREEN^®^ kit than the Progen kit, while the sensitivity was as good as for the Progen kit.

## 1. Introduction

Hantaviruses, recently renamed Orthohantavirus, are globally distributed and divided into two groups; “The New World” viruses, causing Hantavirus Pulmonary Syndrome (HPS), and “The Old World” viruses, causing Hemorrhagic Fever with Renal Syndrome (HFRS). Puumala virus (PUUV) is the predominant type in Europe, with a majority of cases in Scandinavia and northern Europe. PUUV causes a mild form of HFRS, also called nephropathia epidemica (NE), with a mortality ranging from 0.1% to 0.4% [1,2,3]. Less prevalent in Europe are Dobravavirus (DOBV) and Seoul hantavirus (SEOV). DOBV causes moderate to severe HFRS with a mortality rate of 5%–10%. In addition, in recent years, SEOV has been increasingly detected in wild rats in France, Belgium, the United Kingdom and the Netherlands [4].

Enzyme-linked immunosorbent assays (ELISA) are the method of choice for serodiagnosis of PUUV. The University Hospital of Leuven is the National Reference Center for the detection of Hantavirus infections in Belgium. Currently, the PUUV IgM/IgG ELISA test of Progen is used as a screening test. In cases where other hantaviruses are suspected, based on travel history, an “in house” developed PCR is performed. However, the detection of PUUV viral RNA is only successful during the first days of illness, emphasizing the importance of antibody detection for the diagnosis of this infection.

The aim of this study is to compare the performance characteristics of the Hantavirus (Puumala) IgM/IgG ELISA of Progen and the Hantavirus (Puumala) IgM/IgG ELISA RIDASCREEN^®^ kit (Darmstadt, Germany). Currently, the Progen kit is used in our laboratory. However, the production of this assay will stop in the near future, leading to a necessary search for an alternative assay. To our knowledge, four assays are on the market: Progen, RIDASCREEN^®^, IBL Puumalavirus (Tecan, Hamburg, Germany) and Reagena (Toivala, Finland). The Reagena Puumala assay was validated in our laboratory in 2014 [5].

Both Progen and RIDASCREEN^®^ ELISA are based on the detection of the PUUV nucleocapsid protein (Np), which dominates the antibody response. Both Progen and RIDASCREEN^®^ IgM ELISA are based on a direct-coated *E. coli*-expressed aminoterminal (aa 1–119) PUUV Np.

## 2. Materials and Methods 

A total of 130 patient serum samples from the University Hospital of Leuven and the Institute of Tropical Medicine (ITM) (Antwerp, Belgium) were retrospectively analyzed. All samples were tested with the RIDASCREEN^®^ kit (IgG and IgM: < 16 negative, 16–20 borderline, > 20 positive) and the Progen kit (IgG: < 1 negative, 1–1.5 borderline, > 1.5 positive; IgM: < 1 negative, 1–2 borderline, > 2 positive) using the BEP^®^ III (Diasorin, Sallugia, Italy) platform. When results were discordant, additional testing by immunoblotting (recomLine HantaPlus IgM/IgG, Mikrogen, Neuwied, Germany) was conducted. The reference result, as shown in Table 1, was determined by either concordant test results with both ELISA test kits or the result of a confirmatory immunoblot (IB) for discrepant ELISA test results.

To evaluate sensitivity, 44 samples from patients with an acute PUUV infection (IgM positive) and another 24 samples from patients with a past infection (exclusively IgG positive), based on the result obtained with the Progen kit, were selected. 

To assess specificity, 7 samples with false borderline Progen IgM (no seroconversion occurred in follow-up sample) and 25 negative samples for IgG and IgM were included. Cross-reactivity was assessed with 30 serum samples from patients with a cytomegalovirus (CMV), Epstein Barr virus (EBV) infection or with rheumatoid factor (RF) positivity; i.e., 10 samples positive for CMV IgM, 10 EBV VCA IgM positive samples and 10 RF positive samples. CMV and EBV were analyzed on Architect (Abbott Diagnostics, Lake Forest, IL, USA) (EBV ΔOD/CO ≥ 1, CMV ≥ 6 AU/mL). RF positivity was analyzed with Rheumatoid Factor (Beckman Coulter, CA, USA) on an IMMAGE^®^800 Immunochemistry System (Beckman Coulter) (> 40 IU/mL). All 30 samples were tested with the Progen and RIDASCREEN^®^ kit and were confirmed, in cases of discordance for IgG/IgM or positivity for IgM, using Mikrogen recomLine IB.

The Cohen kappa coefficient was calculated according to qualitative results—positive, borderline, negative—to measure agreement between the Progen and RIDASCREEN^®^ kit. Statistical analyses were performed using Analyse-it Version 5.11 Software (2008, Leeds, United Kingdom) 

## 3. Results

From the 130 samples, 21 showed discordance for PUUV IgM between the Progen and RIDASCREEN^®^ kit. Table 1 shows an overview of these results. Substantial agreement was observed with a Cohen kappa coefficient of 0.76 (95% CI 0.72 0.80). The sensitivity to detect an acute hantavirus infection was 100% for the RIDASCREEN^®^ test. The specificity of the IgM assay was, respectively, 77.0% (95% CI 73.15% 80.9%) and 94.3% (95% CI 89.6% 99%) for the Progen and RIDASCREEN^®^ kit. Notably, the seven false borderline samples with Progen were all negative with IB. Two out of the 10 samples from patients with a CMV infection showed a borderline IgM result with Progen, and one tested positive with RIDASCREEN^®^. IB was also negative for PUUV in these samples. For the 10 samples from patients with a primary EBV infection, four showed discordant IgM results. Three were borderline with Progen and negative with the RIDASCREEN^®^ kit. One was positive in Progen and negative in RIDASCREEN^®^. Furthermore, two samples showed positivity for IgM with both assays and were negative on IB. Four out of the 10 RF positive samples showed borderline results with the Progen kit, but negativity with RIDASCREEN^®^. 

Considering the PUUV IgG PUUV detection test, for the 130 analysed patient sera, 11 were discordant between the two assays. Substantial agreement was observed, with a Cohen kappa coefficient of 0.90 (95% CI 0.86 0.94). The sensitivity for the Progen and RIDASCREEN^®^ kit was, respectively, 98.3% (95% CI 93.4% 100%) and 100%. Progen showed one negative sample, not close to the cut-off of positivity, with a positive result on IB. 

The specificity for the Progen and RIDASCREEN^®^ kit was, respectively, 93.0% (95% CI 93.4% 100%) and 94.4% (95% CI 89.7% 99.10%). Overall, few discordances for IgG were noted between both assays. Seven samples were positive with Progen and negative in IB. Four samples were positive with RIDASCREEN^®^ and negative in IB, with three samples high above the cut-off of positivity.

## 4. Discussion

Our evaluation reveals that the RIDASCREEN^®^ IgG/IgM ELISA kit has a very good sensitivity and specificity to diagnose PUUV infections compared to the Progen test.

Both the Progen and RIDASCREEN^®^ test are based on the direct-coated *E. coli*-expressed aminoterminal PUUV Np. Although both assays are based on the same testing method, the specificity for IgM was a lot higher for the RIDASCREEN^®^ kit (94.3% versus 77.0%). Excellent specificity is usually at the expense of sensitivity, but for the RIDASCREEN^®^ test, this is not the case. Our results for the Progen kit were comparable with the study of Muyldermans et al., who found a sensitivity for IgM of 100% and a specificity of 73.2%. As mentioned, this study was also performed in our laboratory. Based on the results of this study, the Reagena assay kit was not implemented, because of the lower sensitivity compared to the Progen assay [5]. No publications were found on the IBL Puumalavirus assay.

Polyclonal IgM stimulation is a well-known problem in several infections. Therefore, we selected a set of challenging samples to test the specificity of both assays. It can be expected that specificity in a routine setting will be higher than in this study with a bias to false positivity. With the Progen kit, many of these challenging samples tested borderline in the IgM assay but were negative with the RIDASCREEN^®^ assay.

To the best of our knowledge, this is the first study conducted with the newly developed RIDASCREEN^®^ Hantavirus Puumala IgG/IgM ELISA kit showing an excellent sensitivity and specificity for IgM and IgG. It could be stated that the RIDASCREEN^®^ assay has at least the same performance as the Progen assay. Based on these results, the RIDASCREEN^®^ assay was implemented in our laboratory. Although the results of these assays are satisfying, future studies can focus on the performance of the IBL Puumalavirus assay.

## Figures and Tables

**Table 1:** Results of Puumala virus IgM and IgG detection on 130 serum samples with Progen and RIDASCREEN^®^ ELISA and Mikrogen immunoblot.

Samples (*n*)	Progen						Ridascreen						Confirmation immunoblot Mikrogen						Reference			
	IgM			IgG			IgM			IgG			IgM (*n* =21)			IgG (*n* = 11)			IgM		IgG	
	Pos	Bor	Neg	Pos	Bor	Neg	Pos	Bor	Neg	Pos	Bor	Neg	Pos	Ind	Neg	Pos	Ind	Neg	Pos	Neg	Pos	Neg
**Sensitivity**																						
**Acute NE (*n* = 44)**	44			36	3	5	43		1	36	2	6			1 *	3 **		2 ***	43	1	37	7
**Past NE (*n* = 24)**			24	24					24	21	1	2						3^$^		24	22	2
**Specificity**																						
**Potentially false positive IgM (*n* = 7)**		7				7		1	6	2		5			7			2		7		7
**Seronegative Progen PUUV (*n* = 25)**			25			25		1	24			25			1					25		25
**Potentially crossreacting (*n* = 30)**																						
**Acute EBV (*n* = 10)**	3	3	4			10	2		8			10			6					10		10
**Acute CMV (*n* = 10)**		2	8			10	1		9			10			2					10		10
**Positive RF (*n* = 10)**		4	6			10			10	1		9			4			1		10		10

The reference result was determined by either concordant test results with both ELISA testkits or the result of a confirmatory immunoblot for discrepant ELISA test results (CMV: cytomegalovirus; EBV: Epstein Barr virus; NE: nephropathia epidemica; PUUV Puumala virus; RF: rheumatoid factor); * positive for IgM in Progen, negative in Ridascreen; ** one negative for IgG in Progen, borderline in Ridascreen; one positive for IgG in Progen, borderline in Ridascreen; one borderline for IgG in Progen, positive in Ridascreen; *** one borderline for IgG in Progen, negative in Ridascreen; one negative for IgG in Progen and positive in Ridascreen; ^$^ two positive for IgG in Progen, negative in Ridascreen; one positive for IgG in Progen, borderline in Ridascreen.

## References

[1] Avsic-Zupanc T., Saksida A., Korva M. (2019). Hantavirus infections. Clin. Microbiol. Infect..

[2] Sargianou M., Watson D.C., Chra P., Papa A., Starakis I., Gogos C., Panos G. (2012). Hantavirus infections for the clinician: From case presentation to diagnosis and treatment. Crit. Rev. Microbiol..

[3] Hjertqvist M., Klein S.L., Ahlm C., Klingstrom J. (2010). Mortality rate patterns for hemorrhagic fever with renal syndrome caused by Puumala virus. Emerg. Infect. Dis..

[4] Verner-Carlsson J., Lõhmus M., Sundström K., Strand T.M., Verkerk M., Reusken C., Yoshimatsu K., Arikawa J., de Goot F.V., Lundkvist Å. (2015). First evidence of Seoul hantavirus in the wild rat population in the Netherlands. Infect. Ecol. Epidemiol..

[5] Muyldermans A., Lagrou K., Patteet S., Van Esbroeck M., Van Ranst M., Saegeman V. (2014). Comparison of two automated immunoassays for the determination of Puumalavirus IgM and IgG. J. Clin. Virol..

